# Quantification of interacting cognate odorants with olfactory receptors in nanovesicles

**DOI:** 10.1038/s41598-017-16997-9

**Published:** 2017-12-13

**Authors:** Marta Sanmartí-Espinal, Patrizia Iavicoli, Annalisa Calò, Marta Taulés, Roger Galve, M. Pilar Marco, Josep Samitier

**Affiliations:** 1 IBEC - Institute for Bioengineering of Catalonia-Barcelona Institute of Science and Technology (BIST), Barcelona, Spain; 2 Department of Engineering: Electronics, University of Barcelona, Barcelona, Spain; 3 Centres Científics i Tecnològics, University of Barcelona, Barcelona, Spain; 4 Nb4D – Nanobiotechnology for Diagnostics, IQAC-CSIC, Barcelona, Spain; 5 Centro de Investigación Biomédica en Red en Bioingeniería Biomateriales y Nanomedicina (CIBER-BBN), Madrid, Spain

## Abstract

This study aims to improve our understanding of the interaction between olfactory receptors and odorants to develop highly selective biosensing devices. Natural nanovesicles (NVs) from *Saccharomyces cerevisiae*, ~100 nm in diameter, carrying either the human OR17-40 or the chimpanzee OR7D4 olfactory receptor (OR) tagged with the c-myc epitope at their N-terminus, are presented as model systems to quantify the interaction between odorant and olfactory receptors. The level of expression of olfactory receptors was determined at individual NVs using a novel competitive ELISA immunoassay comparing the values obtained against those from techniques involving the solubilization of cell membrane proteins and the identification of c-myc-carrying receptors. Surface Plasmon Resonance (SPR) measurements on L1 Biacore chips indicate that cognate odorants bind to their Ors, thereby quantifying the approximate number of odorants that interact with a given olfactory receptor. The selectivity of OR17-40-carrying NVs towards helional and OR7D4-carrying NVs towards androstenone has been proven in cross-check experiments with non-specific odorant molecules (heptanal and pentadecalactone, respectively) and in control receptors.

## Introduction

The animal nose is capable of distinguishing between trillions of smells at very low concentrations^1,2^. As a result, mammalian olfactory receptor-based sensing devices have shown sensitivity to a variety of odorant molecules^3,4^. Basically, the biologically active part in these devices is made up of olfactory receptors immobilized^5^ on functionalized surfaces. Because olfactory receptors are G-coupled membrane proteins, their integration into a lipid envelope is considered necessary to ensure their tertiary structure and functionality is retained^6^. In particular, the isolation of natural vesicles from a cell source where mammalian olfactory receptors have been heterologously expressed provides an environment that is as native as possible for the receptor proteins and already proven to be successful in the design of the active part of bioelectronic noses^7^.

In order to realize practical biomolecular devices based on OR proteins embedded in natural vesicles, protein expression level control in vesicles as well as verification of their functionality need to be strictly managed.

In this work, we determine the expression level of two different olfactory receptors: the human OR17-40 and the chimpanzee OR7D4. Both are tagged with the c-myc epitope at their N-terminus in nanovesicles (NVs) from *Saccharomyces cerevisiae*. A proof of concept of the specific binding of helional by OR17-40-carrying vesicles, and of androstenone by OR7D4-carrying vesicles is presented, based on Surface Plasmon Resonance (SPR)^8^ Biacore T100 measurements. By comparing the Biacore signal obtained upon binding of odorant onto olfactory receptors to that obtained upon NVs immobilization on sensor chips, and using the number of olfactory receptors in a NV, we were able to estimate for the first time the approximate number of odorant molecules interacting with an olfactory receptor to be between 100 and 1000. Furthermore, this is a first step towards developing an array^9^ for high throughput recognition of multiple odorant^10–12^, which would require the use of a set of different specific OR-carrying NVs.

## Results

### Characterization of NVs carrying olfactory receptors in solution and immobilized on the substrate

**Figure 1:**
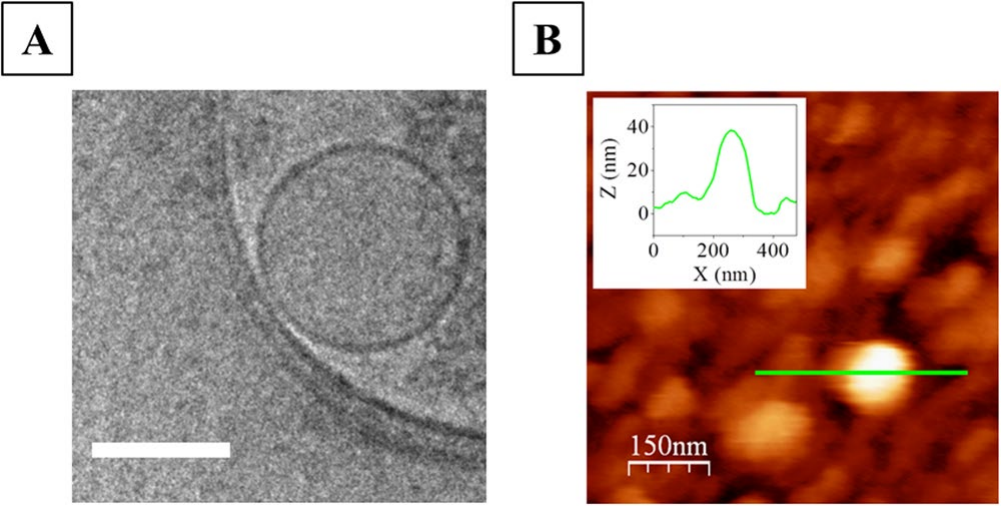
2D Cryo-EM image of an individual NV carrying the OR17-40 olfactory receptor as model NV. Scale bar is 100 nm (**A**). Tapping-mode AFM image of a NV deposited onto a SAM-COOH functionalized gold chip (**B**) and its corresponding topographical profile (B, inset).

Figure 1 shows a typical Cryo-EM (Fig. 1A) and a tapping-mode AFM (Fig. 1B) image of an individual NV in buffer solution. Suspended NVs appear as spheres with an average size of around 100 nm in Cryo-EM images (see Fig. 1A). When deposited onto a SHC_11_PEG_6_OCH_2_COOH self-assembled monolayer functionalized gold surface (SAM-COOH), the vesicle surface flatens, exhibiting an aspect ratio of *h/w* ~0.15, with *h* being the vesicle’s height and *w* its width, as determined by statistical analysis of the topographical profiles from AFM images. The SAM-COOH functionalized gold surface was taken as a reference to evaluate the shape of the NVs after immobilization on a hydrophilic and negatively charged surface^10^ such as the Biacore L1 chip (see Fig. S3). As expected, the surface rendered the identification impossible using conventional AFM. It is reported in the literature that AFM images of a dextran matrix before and after liposome (~100 nm diameter size) incubation, performed with conventional AFM tips, did not show any significant difference^13^. On SAM-COOH functionalized gold substrates, we achieved a maximum NV surface coverage of ~15% by controlling concentration and flow-time parameters in SPR devices.

The geometry of the flattened vesicles from AFM images was consistent with their size in solution which suggests that during the deposition process vesicle volume was preserved. In our experimental conditions, we found no evidence of either full vesicles collapsing or opening in combination with subsequent formation of lipid bilayer patches.

### Assessment of the expression level of OR17-40 and OR7D4 in the total membrane fraction of *Saccharomyces cerevisiae*

**Figure 2:**
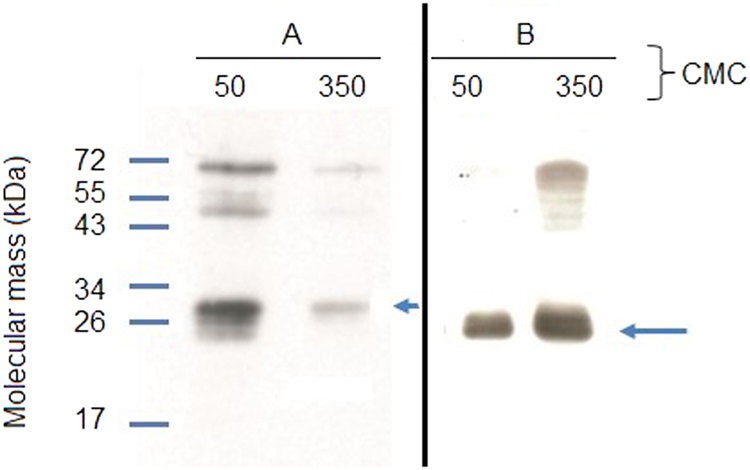
Western Blot of eluates of (**A**) purified c-myc-OR17-40 and (**B**) c-myc-OR7D4 after solubilization of the membrane fraction of *Saccharomyces cerevisiae*. 450 μg of c-myc-OR membrane fractions were solubilized using FC14 at 50 or 350 CMC.

Total membrane fraction of *Saccharomyces cerevisiae* yeast cells carrying heterologously expressed human c-myc-OR17-40 olfactory receptors or chimpanzee c-myc-OR7D4 olfactory receptors were prepared by means of cell disruption. The olfactory receptor content in the total membrane fraction was determined after solubilization of all the proteins from yeast lipid membranes in fos-choline 14 (FC14) detergent and purification of the cmyc-ORs onto an affinity column with an anti-c-myc antibody (see Materials and Methods). Subsequent absorbance quantification of the eluted purified receptors gave (0.0030 ± 0.0005) g c-myc-OR17-40/g total proteins and (0.008 ± 0.002) g c-myc-OR7D4/g total proteins. Western blotting (WB) was also performed to check protein quality in these eluates. Figure 2 shows the WB of a c-myc-OR17-40 purified sample (Fig. 2A and Supplementary Figure S7A) and of a c-myc-OR7D4 purified sample (Fig. 2B and Supplementary Figure S7B). An immunoreactive band was observed at about 30 kDa for c-myc-OR17-40 and at about 26 kDa for c-myc-OR7D4, which is consistent with the molecular weight of the monomeric form of the receptors (see the blue arrow in Fig. 2). Bands corresponding to oligomeric forms of the receptors were also observed. The higher intensity of the monomeric OR band with 50 CMC compared to 350 CMC for the c-myc-OR17-40 eluate may be due to a modification of the receptor conformation that reduces accessibility of the c-myc epitope or its affinity or induces the masking of the c-myc epitope by the detergent. As regards the c-myc-OR7D4 eluate, neither the 50 CMC nor the 350 CMC conditions induced any such differences.

### Determination of OR expression levels in individual NVs

Because the expressed ORs of interest carry the c-myc peptide on their N-terminus, we built a calibration curve by ELISA immunoassay using reference proteins carrying the c-myc peptide. These reference proteins were dissolved in a solution of SSTR2-carrying NVs as the closest negative control for OR-carrying nanovesicles. Next, the OR-carrying NV absorbance signal was compared against reference protein calibration curves for direct quantification of the number of receptors per NV.

**Figure 3:**
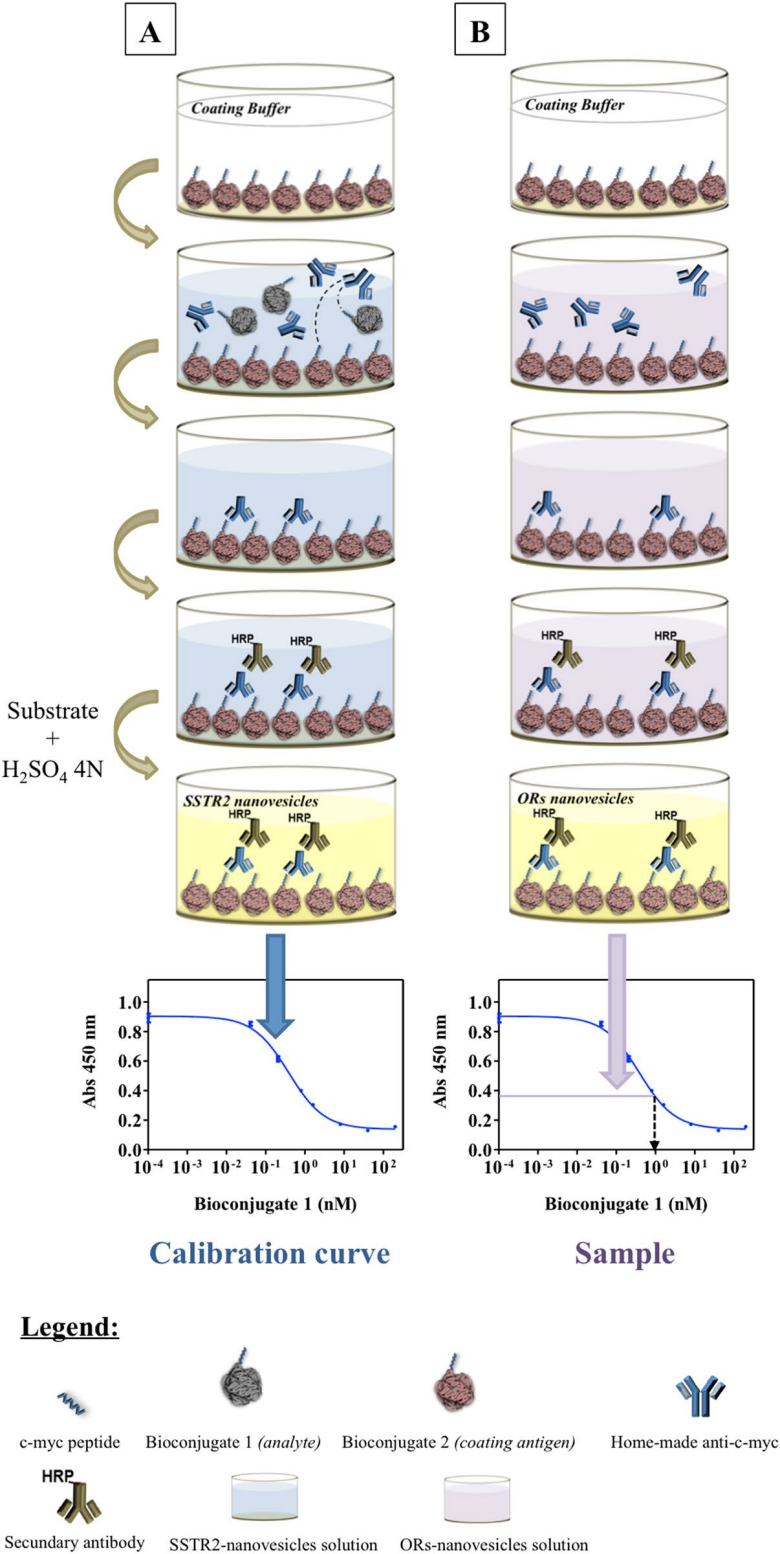
Scheme of the competitive ELISA assay. (**A**) Calibration curve generation using the c-myc-Bioconjugate 1 in an SSTR2-carrying NV solution; (**B**) the C-myc-OR-carrying NV signal (**B**) is compared against the calibration curve performed in (**A**) to provide the concentration of c-myc-ORs.

Basically, reference proteins and the coating antigen are well-known proteins to which the c-myc tag peptide is covalently attached (c-myc-bioconjugates) achieving picomolar sensitivity in the competitive assay (see Fig. 3).

The ELISA experiment was performed at different NV concentrations (4.2·10^10^ to 12.6·10^10^ NV/mL, 25–45 μg/mL of total protein, see Supplementary Figures S2 and S3 and Table S1).

**Figure 4:**
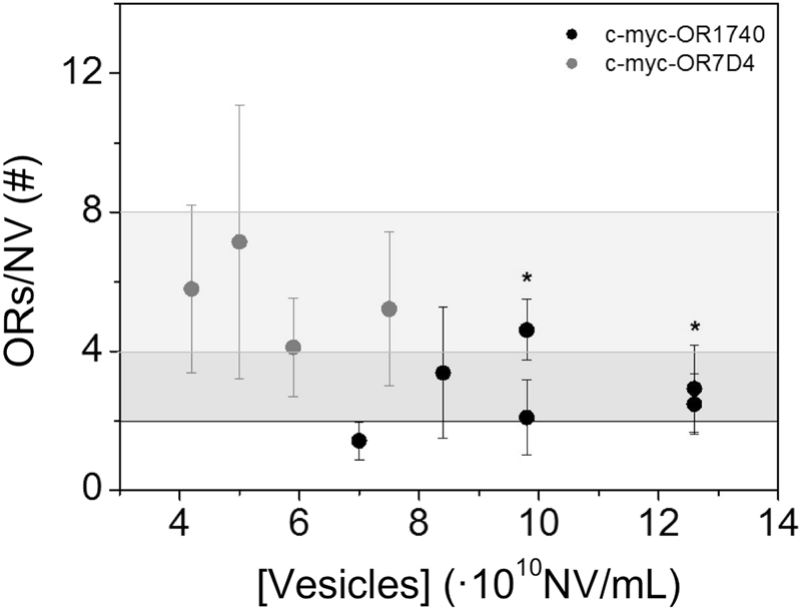
Number of receptors per NV (ORs/NV) as a function of NV concentration. The black dots correspond to c-myc-OR1740-NV and the grey dots correspond to c-myc-OR7D4-NV. The dark grey area shows (3 ± 1) c-myc-OR1740/NV and the light grey area shows (6 ± 2) c-myc-OR7D4/NV. Results from repeat experiments are shown with an asterisk (*).

Figure 4 shows the number of receptors per NV at each concentration obtained through direct measurements of the number of receptors per mL divided by the number of NV per unit volume, as determined by Nanoparticle Tracking Analysis (NTA). We obtained 3 ± 1 c-myc-OR17-40/NV as the weighted mean of six samples at four different concentrations and 6 ± 2 c-myc-OR7D4/NV as the weighted mean of four samples at four different concentrations. Considering the surface area of a 100 nm diameter NV, this number can be converted into a density of ~150 ORs/µm^2^. This corresponds to a mean distance between receptors in the order of approximately 100 nm for both the OR17-40- and OR7D4- carrying NVs (see Supplementary Table S2). During the process of nanovesicle preparation, membrane fractions break down into nanofragments which reclose into nanovesicles. No fusion of nanofragments has ever been observed in previous experiments. Nanovesicle size only decreases during the process of sonication until a plateau is reached (personal data), and, therefore, association of upside-down fragments is highly unlikely. Because of the differential composition between inner and outer lipid layers, it can be speculated that after sonication fragments tend to reclose following the same orientation as they originally had in the cell. The ELISA assay only quantifies c-myc epitopes outside the NVs, and, therefore, all ORs on the NVs are likely to be taken into account with this technique.

Values from direct measurements on the NVs can be compared with estimates which we obtained from the protein fraction of expressed OR in the lipid membrane before the actual NVs were obtained. Considering the molecular weight of the receptor (34 kDa), there are 5,3.10^16^ OR/g Total Protein. Since Figure S2C provides a correlation between the amount of cmyc-OR1740-nanovesicles and the amount of TP, we can estimate 19 ± 3 ORs/NV. In the same way, for c-myc-OR7D4-nanovesicles, we calculated 0.008 g OR/g Total Protein using the solubilization/purification technique. Considering the molecular weight of the receptor (26 kDa), there are 14,1.10^16^ OR/g Total Protein. Since Supplementary Figure S2 provides a correlation between the amount of cmyc-OR7D4-nanovesicles and the amount of TP, we obtained 84 ± 21 ORs/NV. Nevertheless, the quantification ratio between OR7D4 and OR1740 obtained using the direct nanovesicle ELISA method was 3 to 6 times lower than that estimated through the protein purification technique. However, this difference is relatively small considering that two very different methods were used to estimate the number of ORs/NV and could therefore be considered as the lowest and highest limit of OR quantification.

### Assessment of OR17-40 and OR7D4 binding functionality by Surface Plasmon Resonance (SPR)

**Figure 5:**
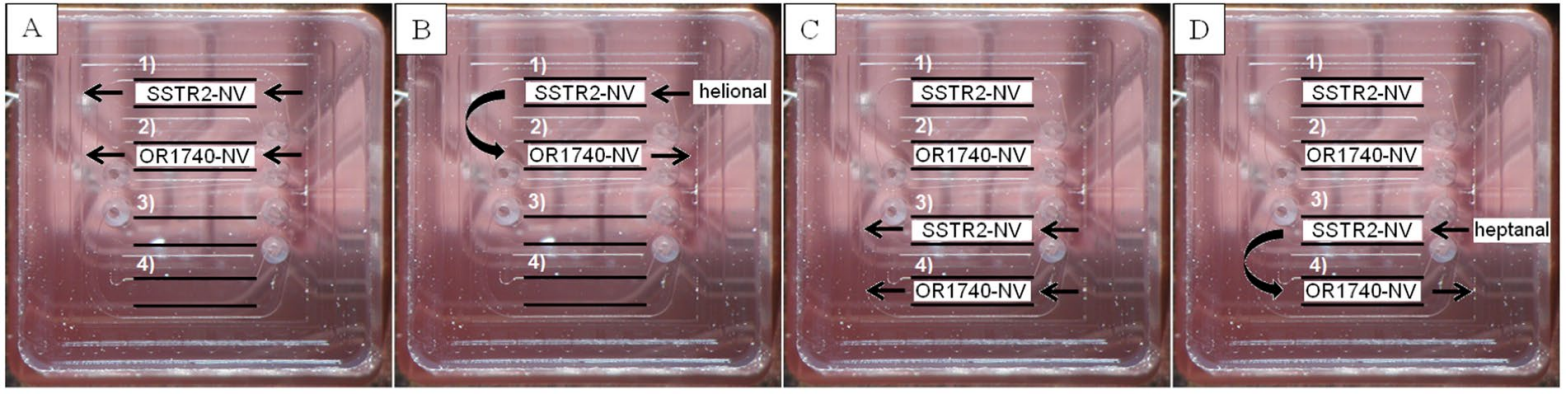
Optical images of the Biacore T100 flow cell. In the SPR assay, a solution of nanovesicles and control nanovesicles is immobilized in two different channels (**A**). Then, three helional solutions at different concentrations are sequentially flowed across the cell (**B**). A solution of nanovesicles and control nanovesicles is again immobilized in the two remaining channels (**C**) and control heptanal solutions sequentially flowed through the two channels (**D**).

In order to verify the binding activity of the olfactory receptors integrated in NVs towards odorants, we developed an SPR-based assay that uses double-reference analysis^14^. The experiment is described in Fig. 5 for assessment of the OR17-40 response towards odorants. Double reference analysis was applied for measuring the device response to both the specific odorant helional^15^ (see Fig. 5B) and the control odorant heptanal (see Fig. 5D). NV solutions containing the OR17-40-nanovesicles or the SSTR2 control receptors-nanovesicles were captured in equivalent amounts (capture level ~1000 RU, see Materials and Methods) in two different channels of the device (Fig. 5A). Next, a solution of helional at different progressively increasing concentrations (2, 3, 5 µM) was flowed across SPR channels (see Fig. 5B). Signals from either the SSTR2-NV (reference channel) or OR17-40-NV channel were first obtained by subtracting the signals provided with the running buffer (blank) (see Materials and Methods) from those obtained with helional. The result obtained from SSTR2-NV was then subtracted from that of OR17-40-NV. The same procedure was repeated for the control odorant heptanal (Fig. 5C and D). The two odorant solutions were flowed independently through the same chip, first through channel 1 and 2 and then through channel 3 and 4 to avoid measurement dispersion due to heterogeneities among different Biacore chips.

The same procedure was performed with OR7D4-NV to assess its binding functionality against androstenone^16^ using OR17-40-NV as control NV and pentadecalactone as control odorant.

**Figure 6:**
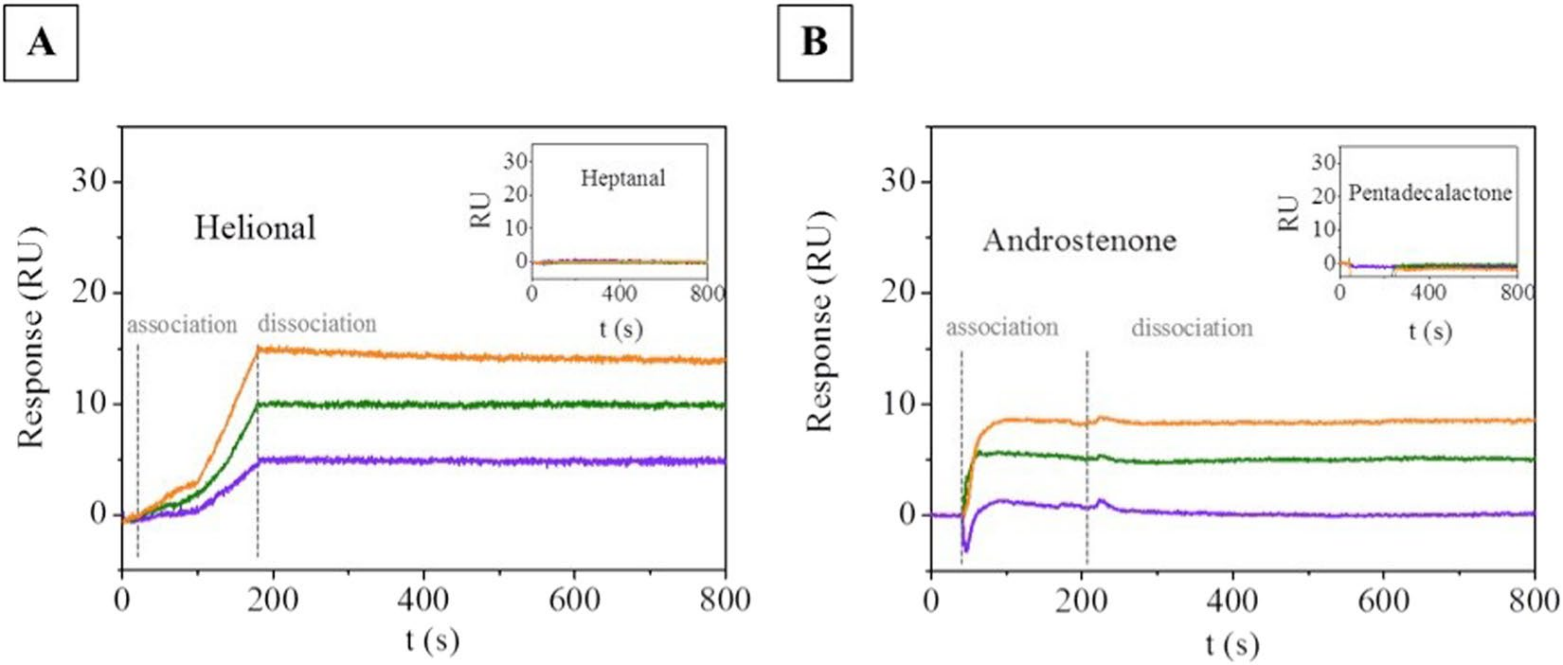
(**A**) Double-reference SPR sensorgrams obtained by flowing solutions of helional at 2 µM (data in violet), 3 µM (data in green) and 5 µM (data in orange) onto a L1 chip with OR17-40-NV (flow rate: 60 µL/min). The association phase was followed for 180 s and the dissociation phase was followed for 620 s. Inset: Corresponding sensorgrams obtained by flowing through the same chip heptanal solutions at the same concentrations. (**B**) Double-subtracted SPR sensorgrams obtained by flowing solutions of androstenone at 1 µM (data in violet), 5 µM (data in green) and 10 µM (data in orange) onto a L1 chip with OR7D4-NV (flow rate: 60 µL/min).

Figure 6 shows the signal arising from the OR17-40-NV (Fig. 6A) and from the OR7D4-NV (Fig. 6B) after the double subtraction vs. helional and androstenone. It shows the shift of SPR dip (minimum of the SPR angle) registered as resonance units (RU) vs. time (sensorgram) due to the application of helional at three different concentrations (2, 3, 5 µM). No change in RU signal was observed when the unspecific odorant heptanal was applied (see the inset in Fig. 6A). Figure 6B shows the double-referenced sensorgram resulting from the application of androstenone at three different concentrations (1, 5, 10 µM) through OR7D4 and its respective reference. Our results show that both ORs-carrying NVs responded to the corresponding specific odorant in a concentration-dependent manner, exhibiting selectivity towards the different odorant molecules. In OR7D4-NV, we found a similar behavior vs. the pentadecalactone control odorant. To verify that the choice of control NV did not significantly affect the SPR measurement, we also assessed the OR17-40-NV binding functionality vs. helional with the OR7D4-NV control (see Supplementary Fig. S6).

It is worth noting that odorant molecule concentration in the SPR measurements was low because the device detected the lowest possible concentration of the odorant. Due to the odorant’s low molecular weight, SPR measurements resulted in a low signal, close to the lowest detection limit of the SPR equipment. Furthermore, in OR7D4-NV, binding functionality assessment by SPR was also critical for high androstenone concentrations (>20 µM) due to the limited odorant solubility in the running buffer. This, coupled with the highly non-ideal behavior of the SPR response upon odorant injection, made it impossible to determine the equilibrium dissociation constant. In fact, deviations from the ideal were found in all the measurements that never reached the steady state during the association time (see the association part of Fig. 6A). Moreover, these SPR sensorgrams exhibited very slow, or no dissociation process at all, upon completion of the odorant injection (see the dissociation part of Fig. 6A and B).

**Figure 7:**
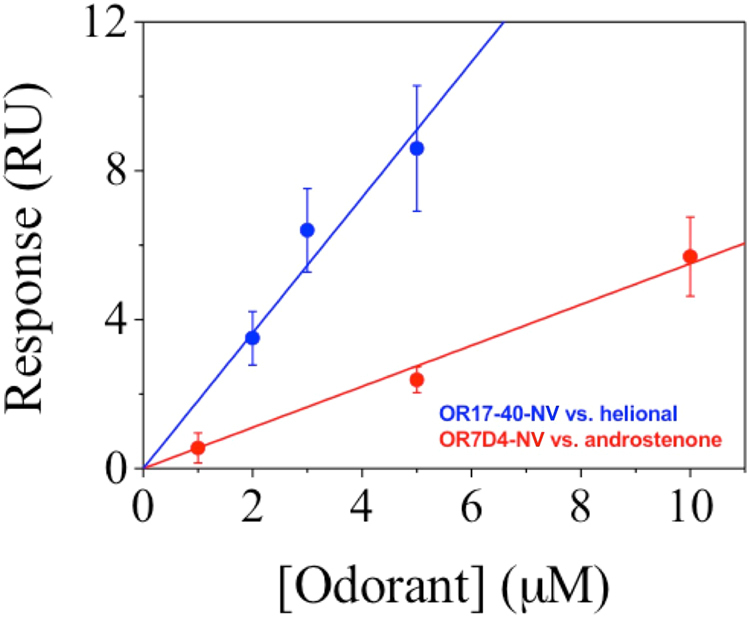
SPR response of OR17-40-NV vs. helional concentration (data in blue) and of OR7D4-NV vs. androstenone concentration (data in red). The corresponding linear fits give: y = 1.82·x (blue line) and y = 0.55·x (red line).

In Fig. 7 we report the evolution of the SPR signal corresponding to the (t = 900 s) of SPR curves vs. the odorant concentration. The minimum odorant concentration (LOD) that we detected with this method was 0.3 µM helional and 0.9 µM androstenone (see Materials and Methods) in agreement with the results reported by J.D. Mainland *et al*.^17^. The linear fits in Fig. 7 yielded a sensitivity of 1.8 RU/μM for helional and 0.6 RU/μM for androstenone in the dose response range studied.

The corresponding linear fit for the plot of SPR signal vs. odorant concentration performed with OR17-40-NV using OR7D4-NV as control NVs yielded a detection limit of 0.4 µM helional and a sensitivity of 1.2 RU/μM (see Supporting Information, S6). This fact suggests that the choice of the control NS does not noticeably affect the calibration parameters of the SPR signal vs. concentration curve. The 2-3 times higher sensitivity of OR17-40-NV towards helional compared to that of OR7D4-NV towards androstenone could suggest a higher affinity constant of the OR17-40 receptor for its specific odorant, especially if we consider that other parameters affecting the SPR signal evolution vs. concentration (such as NV deposited mass) remained constant during the experiment.

## Discussion

A good strategy to develop biomimetic sensors based on olfactory receptors consists in expressing the receptor proteins at the surface of natural cells used as the source for NV production. This strategy keeps the receptors embedded in the original membrane, i.e., a lipid bilayer with other membrane proteins or anchored proteins, which simulates the natural environment maintaining these proteins functional and structurally stable. However, the quantification of the number of receptors present in the vesicles, once prepared, and the verification of their ability to capture specific odorant molecules, are key issues. In this work, we have addressed these issues thoroughly. Using a competitive ELISA immunoassay, we determined the number of human OR17-40 and chimpanzee OR7D4 receptors in NVs from *Saccharomyces cerevisiae* yeast cells where the receptors had been heterologously expressed. By means of the SPR technique, we proved that the expressed receptors specifically bind to the helional and androstenone odorants, respectively.

**Figure 8:**
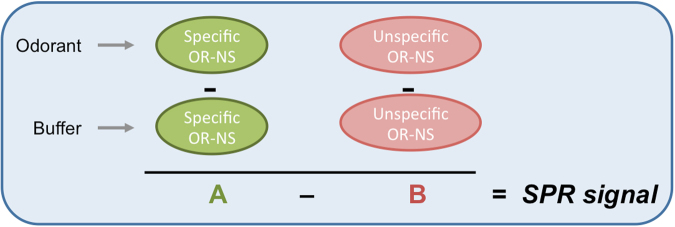
Scheme representing the SPR signal obtained by the SPR based assay presented that uses double-reference analysis.

For both systems, *OR7D4-androstenone* and *OR1740-helional*, the SPR signal is obtained after the subtraction of the blank and of the control nanovesicles signals (Fig. 8). This double-reference analysis is necessary in order to minimize the effect of non-specific adsorption of hydrophobic odorant molecules to the lipid component of liposomes, and the effects of buffer change on the refractive index of the SPR experiments.

Our results indicate that ORs display a binding capacity when prepared in NVs from yeast membranes. As shown, the curvature of the association part for OR17-40 in Fig. 5A is far from ideal which indicates rather complex association kinetics^18^. Furthermore, the association profile suggests a slow association rate constant (k_a_) between OR17-40 and helional. In OR7D4-NV, the association profile suggests a faster association rate constant (k_a_) because a steady state was reached within the same association time. In contrast, the sensorgram dissociation phase of odorants from both ORs-NVs did not show any curvature. This unexpected behavior was not the result of the double reference method used to analyze the data, but rather appeared to indicate a strong stability of the protein-ligand interaction in the NVs *in vitro*. This behavior differs from that found by Cook *et al*.^19^ in solubilized ORs, which displayed standard association and dissociation phases. Using artificially reconstituted liposomes, Oh *et al*.^20^ also observed a fast dissociation. Similarly, Lee *et al*.^21^ found a fast dissociation rate in experiments performed in human embryonic kidney (HEK)-293 cells expressing olfactory receptors. Hence, neither cellular machinery nor receptor environment appears to impact the OR-odorant ligand dissociation phase. However, the results obtained with SPR must be compared carefully, because a not accurate reference analysis could induce a reduction in the sensorgram signal when the odorant injection finishes.

Usually, in Biacore experiments, the maximum binding signal ratio of the SPR surface can be estimated using the formula

1 $$\frac{Rmax}{Rl}=\frac{Mwa}{Mwl}s$$

Where Rmax is the maximum binding capacity (RU), Rl is the response level of immobilized ligands, Mwa is the molecular weight of analyte, Mwl is the Molecular weight of ligand and S is the number of binding sites per ligand.

Using this approach, the incremental SPR binding signal ratio obtained in presence of the highest odorant concentration is approximately 1/100 (10^−2^) (Supplementary Figure S5 and Fig. 6). Bearing in mind that the weight of each odorant molecule is around 10^−10^ pg. and the weight of each NV is around 10^−4^ pg. From Eq (1) our results would indicate that 10000 (10^–2^/10^−6^) odorant molecules might interact with each NV and hence, between 10^2^ and 10^3^ odorant molecules might interact with each OR. Even considering that the linearity of the equation is not conserved, it seems that we are far from a one-to-one interaction and many odorants interact with a nanovesicle and in consequence few odorants interact with an OR. The difficulties to understand completely the SPR sensorgram (association and dissociation phase) and the low level of SPR signal make it difficult to specify the value. This hypothesis would be consistent with the evidence that ORs recognize their cognate ligands through a network of distinct aminoacid residues disposed in their transmembrane domain, and that additional residues may be also involved in odorant interaction^22,23^.

Although the number of olfactory receptors in NVs was in the low range of a few ORs/NV, a fact that is consistent with the general expression level of recombinant GPCR in cellular systems, NVs preparation together with a careful choice of SPR experimental conditions and data analysis, allowed us to clearly demonstrate the very good specificity of odorant ligand binding. Moreover, the study showed that the level of odorant binding onto ORs depends on odorant concentration in both receptors. A lowest detection limit of odorant binding to its OR was obtained, when compared to other groups^19–21^. The study confirms that ORs embedded in nanovesicles properly bind to their cognate odorants, which opens possibilities for their use as recognition biomaterial for odorant detection at low concentrations.

## Methods

### Chemicals and Material

#### Reagents

In this work, the reagents phosphate-buffered saline (PBS), anti-mouse IgG peroxidase, Tween 20, tetramethylbenzidine (TMB), sodium citrate, 3-[(3-cholamidopropyl)dimethylammonio]-1-propanesulfonate hydrate (CHAPS), dimethyl sulfoxide (DMSO), 4-(2-hydroxyethyl)-1-piperazine-ethanesulfonic acid (HEPES), bovine serum albumin (BSA), triethylene glycol mono-11-mercaptoundecyl ether (PEG_3_-thiol), androstenone, pentadecalactone, and heptanal were purchased from Sigma-Aldrich Chemie GmbH (Germany). Helional was a generous gift from Givaudan-Roure (Dübendorf, Switzerland), courtesy of B. Schilling. Potassium dihydrogen phosphate, disodium hydrogen phosphate, sodium carbonate, sodium hydrogen carbonate, and Tris (hydroxymethyl) aminomethane were purchased from Merck (Darmstadt, Germany). NaOH pellets, sodium chloride, and ethanol absolute were obtained from Panreac Química S.A.U. (Barcelona, Spain). The 2-(2-{2-[2-(2-[2-(11-mercapto-undecyloxy)-ethoxy]-ethoxy)-ethoxy]-ethoxy}-ethoxy (SAM-COOH) was supplied by ProChimia Surfaces Sp. (Sopot, Poland). The FC14 detergent was acquired from Affymetrix Anatrace products (Santa Clara, USA). Nitrocellulose membranes (Hybond^TM^-C extra) were from Amersham (GE Healthcare Europe). The monoclonal anti-c-myc IgG_1_ 9E10 antibody and the antiproteases inhibitors cocktail were obtained from Roche Diagnostics (Mannheim, Germany). Profound TMc-Myc Tag IP/Co-IP kit was supplied by Thermo Scientific (Rockford, IL, USA). In addition, MaxiSorp polystyrene 96-well plates were purchased from Nunc (Roskilde, Denmark). L1 sensor chips were purchased from GE Healthcare Bio-Sciences AB (Uppsala, Sweden). The gold chip for SPR measurements was purchased from GWC Technologies Inc.

#### Buffers and solutions

Buffers used were as follows: phosphate-buffered saline (PBS) 10 mM at pH 7.5, PBST as PBS with 0.05% Tween 20 and PBT as potassium dihydrogen phosphate/disodium hydrogen phosphate (10 mM at pH 7.5) with 0.05% Tween 20. The Tris buffer was at 1 M pH 8 and TBST was 25 mM tris-buffered saline (pH 7.2) and 0.15 M NaCl with 0.05% Tween 20. Coating buffer was 50 mM carbonate/bicarbonate at pH 9.6. Citrate buffer was sodium citrate 40 mM solution at pH 5.5. The substrate solution was 0.01% TMB and 0.004% H_2_O_2_ in citrate buffer. The running buffer used in Biacore experiments was HBSN (25 mM HEPES, 150 mM sodium choride) with 1% dimethyl sulfoxide (DMSO) at pH 7.4. The CHAPS solution used was 20 mM at pH 7.4.

### Equipment/Software

2-D cryo-imaging was obtained using the FEI Tecnai microscope (FEI Company, Eindhoven, Netherlands). The peristaltic pump used was from Ismatec (Glattbrugg, Switzerland) and the Surface Plasmon Resonance RT2005 equipment was from Resonant Technologies GmbH (Germany). Proteins from the different yeast membrane fraction batches were quantified using the Pierce BCA Protein Assay kit (Thermo Scientific, USA) to obtain the Total Protein Concentration (TPC), and eluted c-myc-tagged OR, on a Nanodrop instrument (Labtech, UK). Protein bioconjugates were characterized by MALDI-TOF-MS (matrix assisted laser desorption ionization time-of-flight mass spectrometer) from Bruker Biflex III (Bruker, Karlsruhe, Germany). The washing steps in the ELISA assays were carried out using an ELx405 HT microplate washer (BioTek, Winooski, VT). The software packages used to read and analyze the ELISA results were SpectramaxPlus (Molecular Devices, Sunnyvale, CA) and GraphPad Prism v4.00 (GraphPad Software Inc., San Diego, CA). The olfactory receptor binding experiments were performed on a Biacore T100 instrument, GE Healthcare Bio-Sciences AB. All biosensor data processing and analysis was performed using Scrubber2 software (BioLogic Software). The AFM images were acquired with a MFP-3D AFM (Asylum Research) and analyzed using WSxM software (Nanotech, http://www.nanotec.es/).

### Nanovesicles

#### Nanovesicles preparation

Human olfactory receptor c-myc-OR17–40 (ORL520 in OrDB), chimpanzee olfactory receptor c-myc-OR7D4, and somatostatin receptor subtype 2 (SSTR2) were expressed in different yeast *Saccharomyces cerevisiae* cultures. Membrane fractions were obtained following a procedure described in the literature^11,24^. Prior to use, the stock suspension of membrane fractions was diluted in the corresponding phosphate buffer according to the corresponding method. Further homogenization steps, as well as the characterization of the NVs (size, concentration, etc.), were performed following the protocol described in previous works^10,25^. *ELISA assays*: The stock suspension of the membrane fractions was diluted in PBT at a total protein concentration (TPC) of 60 μg/mL. Subsequently, more PBT buffer was added to this solution until working NV concentrations were reached. *Surface immobilization assays*: Membrane fractions were diluted in PBS at a total protein concentration between 60–85 μg/mL. *Biacore T100 assays:* NV solutions were prepared in PBS at the total protein working concentration of 15 µg/mL following the same protocol.

#### Cryo-electron microscopy (Cryo-EM)

The two-dimensional (2D) imaging of vitrified NV samples was performed on a FEI Tecnai electron microscope (cryo-EM) operating at 200 kV, at a temperature between minus 170 °C and minus 175 °C under low-dose imaging conditions.

#### Nanovesicles immobilization onto a carboxylic functionalized self-assembled monolayer (SAM-COOH) on gold surface

The SAM over the gold SPR sensor chip was formed by immersing the cleaned SPR sensor chip into the 2 mM thiol solution SAM-COOH for 16–20 hours. The unbound thiols were removed by rinsing with absolute ethanol and the substrate dried under nitrogen for subsequent use. The chip was index-matched to a prism and fitted with a 20 μL flow cell connected to a peristaltic pump. NVs immobilization was performed using a solution of 5.59·10^10^ NV/mL (85 μg/mL TPC) at a flow speed of 58 μL/min. The interaction of NVs with the functionalized gold substrates was followed by Surface Plasmon Resonance (Supplementary Information, S1). At the end of the experiment, AFM images were taken to evaluate NVs morphology on the surface.

#### AFM measurements

The AFM images were acquired with a MFP-3D AFM (Asylum Research) in tapping mode in liquid, using Si_3_N_4_ tips coated with Au/Cr with a spring constant of 0.08 N m^−1^ (Olympus cantilevers) at a scan rate of 1 Hz.

### c-myc-OR Protein Quantification after solubilization

#### Proteins solubilization

Fos-choline-14 (FC14) detergent was used for efficient solubilization of ORs, as described by Cook *et al*.^19^ To investigate the critical step of solubilization, a range of detergent concentrations relative to its critical micelle concentration (CMC) were tested for two different concentrations: 10 to 350 CMC, both with the same amount of membrane fraction. Then, the samples were dot blotted onto a nitrocellulose membrane and hybridized with a commercial anti-c-myc mAb. The minimal efficient FC14 concentration (50 CMC) was determined though correlation analysis between the diameter of the dots and the level of solubilization (data not shown). Solubilization of c-myc-OR17–40 and c-myc-OR7D4 from 450 μg of membrane fractions was performed using FC14 detergent (50 or 350 CMC) together with an antiprotease inhibitor cocktail at 4 °C for 3 hours with gentle end-over-end mixing.

#### Olfactory receptor protein quantification

The quantification of solubilized cmyc-ORs was performed using the ProfoundTMc-Myc Tag IP/Co-IP kit. 10 µL of anti c-myc-agarose was delivered into the solubilized c-myc-ORs in spin columns. The incubation was done with gentle end-over-end mixing at 4 °C overnight. To ensure that contaminating proteins were discarded, the number of necessary washes was determined by measuring the protein concentration after each wash. After eight washes with 0.5 mL TBST, the c-myc-tagged proteins were eluted with 4 × 10 μl elution buffer. The eluates were neutralized with 2 μl of Tris buffer pH 9. Proteins were quantified by Nanodrop at 280 nm, by performing 10 measurements on each eluate. Each eluate was analyzed using western blotting with anti-c-myc antibodies (20 μl of eluate, commercial anti-c-myc antibody 1:8000).

### c-myc-OR Quantification per nanovesicle

The method we developed to directly quantify the number of receptors per NV is based on an indirect competitive ELISA immunoassay that is performed on the NV sample (see Materials and Methods). The major benefit of this method is that the quantification of olfactory receptor expression can be performed directly without the need of previous solubilization and purification of the olfactory receptors from their natural lipid environment.

These assays were performed following a novel immunochemical strategy. The specificities of the methodology from a biochemical point of view (description of competitive ELISA assay optimization, design of the antigen/antibody structure, matrix characteristics, physico-chemical buffer conditions, choice of the bioconjugates, optimal working concentrations, choice of the mathematical model for the data analysis) were published in a recent study^26^.

#### Competitive indirect ELISA

Calibration curves in competitive assays were obtained using four different c-myc-bioconjugates (well-known proteins conjugated with the c-myc peptide and well characterized by MALDI-TOF-MS [data not shown]). Three of these were synthesized to be used as analytes and one as coating antigen. First, 96-well plates were coated with the antigen (0.0125 μg/mL in coating buffer) and left at room temperature for four hours. Next, the plates were washed with PBST. All the washing steps consisted of filling up the wells with PBST (300 μL) and immediately aspirating the solutions using a vacuum pump with an automated microplate washer, on four cycles. The analyte solution, i.e., the c-myc-OR-NV solutions (25–45 μg mL^−1^ TPC in PBT) or the c-myc-bioconjugates (200–0.008 nM in presence of SSTR2 nanovesicles) were added. This was followed by the addition of custom-made anti-c-myc monoclonal antibodies (1:160000). Next, the plates were washed again with PBST and the commercial antibody anti-IgG-HRP (1:6000) was incubated. The plates were then washed again with PBST and the substrate solution was added and incubated before the enzymatic reaction was stopped and the absorbances measured at 450 nm (see Fig. 3).

The curves obtained were fitted following a typical four-parameter logistic equation^27^. Using competitive assays, we carried out the c-myc-OR17-40 and c-myc-OR7D4 quantification per NV at different NV concentrations, for c-myc-OR17-40 at 7.0·10^10^–12.6·10^10^ NV/mL with 25–45 μg/mL of TPC, and for c-myc-OR7D4 at 4.2·10^10^–7.5·10^10^ NV/mL with 25–45 μg/mL of TPC (Supplementary Information, S2). The absorbance values of c-myc-OR samples were interpolated in each corresponding c-myc-bioconjugate calibration curve at the same concentration to obtain the concentration of the c-myc tag in solution which is proportional to the number of c-myc-ORs. The data presented correspond to the average of three well replicates. The assay reproducibility was also evaluated for c-myc-OR17-40 (n = 6) and for c-myc-OR7D4 (n = 4).

### Olfactory Receptor binding functionality

Studies were performed at 25 °C using Biacore T100 and L1 sensor chips preconditioned with three pulses of CHAPS for 1 min each at 5 μl/min and three pulses of Isopropanol:NaOH 50 mM (2:3) for 1 min each at 5 μl/min. All solutions were filtered through 0.22 μm porous filter before use.

#### Odorant solubilization

Odorant solutions were prepared in a fume hood. An initial stock solution of either helional or heptanal at 10^−1^ M in DMSO was prepared and then used to prepare the odorant working concentrations (1, 2, 3, 5, or 10 µM) maintaining a constant concentration of 1% of DMSO. For androstenone and pentadecalactone, we prepared a stock solution at 10^−2^ M in DMSO which was used to produce odorant working concentrations of 1, 5, and 10 µM while again maintaining exactly 1% of DMSO. The blank solutions were prepared in the exact same way as the odorant solution, but replacing the odorant with DMSO.

#### Nanovesicles capture

NVs were immobilized on the surface of a L1 Biacore chip whose matrix contained lipophilic groups covalently attached to carboxymethylated dextran. This sensor chip was chosen for its high capacity for direct attachment of lipid vesicles and its stability over time. Since the lipid bilayer structure is retained after attachment, we can study interactions involving transmembrane receptors in a native-like environment. NVs were injected onto L1 sensor chips at 5 µL/min for 30 minutes to achieve capture levels of about 1200 resonance units (RU). A short pulse (1–3 minutes) of 5 mM NaOH was injected onto the deposited nanovesicles to get rid of potential multilayers. BSA (0.1 mg/mL in PBS) was then flowed at 5 µL/min for 5 minutes to block the sensor chip surface from unspecific odorant adsorption (Supplementary Information, S3). Odorant binding assessment at different concentrations was performed on a non-regenerated NV surface.

#### Odorant analysis

At first, the chip was initialized with running buffer solution (HBSN with 1% DMSO) for chip stabilization. Then, different concentrations (1, 2, 3, 5 or 10 µM) of odorants were injected (in order of increasing concentrations either onto the same channel or as separate injections onto different channels) over a period of 3 minutes at a flow rate of 60 µL/min.

#### Data processing and analysis

We followed a double reference system to analyze the responses^28^ based on two control channels: for the c-myc-OR17–40 a channel functionalized with SSTR2-NVs (reference channel) was used, and either helional or heptanal (control odorant) or buffer (blank) were used. Signals from the SSTR2 reference with either odorant or buffer were subtracted from the corresponding signals of the c-myc-OR17-40 channel. For the c-myc-OR7D4 channel a similar procedure was used, with c-myc-OR1740 as the reference, androstenone as odorant, and pentadecalactone as control odorant. The limit of detection (LOD) was estimated to equal the concentration corresponding to the signal of the blank, i.e., that coming from flowing the running buffer through the captured NVs, plus three times its standard deviation. The assay sensitivity was determined as the slope of the linear fit through a plot of RU vs. odorant concentration, in μM. We performed a total of seven experiments for each odorant concentration (OR17-40-NV vs. helional, control NV: SSTR2-NV), four experiments (OR17-40-NV vs. helional, control NV: OR7D4-NV) and twenty experiments (OR7D4-NV vs. androstenone, control NV: OR17-40-NV). The percentage of experiments that gave a response towards the corresponding odorant was 76%, 75% and 35%, respectively, due to the difficulty in preparing the samples and the small signal difference to be observed between the sample channel and the reference channel.

## Electronic supplementary material

Supplementary Information
