# Supplementary material for: Quantification of interacting cognate odorants with olfactory receptors in nanovesicles
Source: Sci Rep. 2017 Dec 13;7:17483. doi: 10.1038/s41598-017-16997-9 (PMC5727543; doi:10.1038/s41598-017-16997-9)
Supplement: Supplementary file 1 — Supplementary Information [file 41598_2017_16997_MOESM1_ESM.pdf]

Quantification of interacting cognate odorants with olfactory receptors in nanovesicles

Marta Sanmartí-Espinal, PhD, Patrizia Iavicoli, PhD, Annalisa Calò, PhD, Marta Taulés, PhD, Roger Galve, PhD, M. Pilar Marco, PhD, Josep Samitier, PhD

Supplementary information

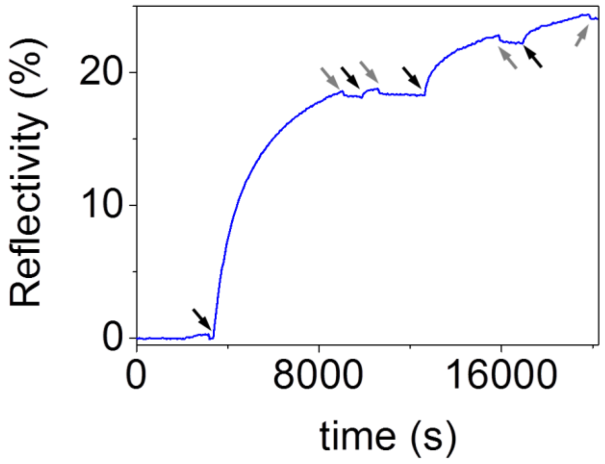

S 1: SPR sensorgram of a nanovesicle solution (concentration = 85 µg/mL of TPC), carrying OR17-40 immobilized onto a SAM-COOH gold sensor chip. Black arrows indicate the injection of nanovesicle solution. Grey arrows indicate the injection of PBS buffer onto the functionalized chip surface after NV immobilization.

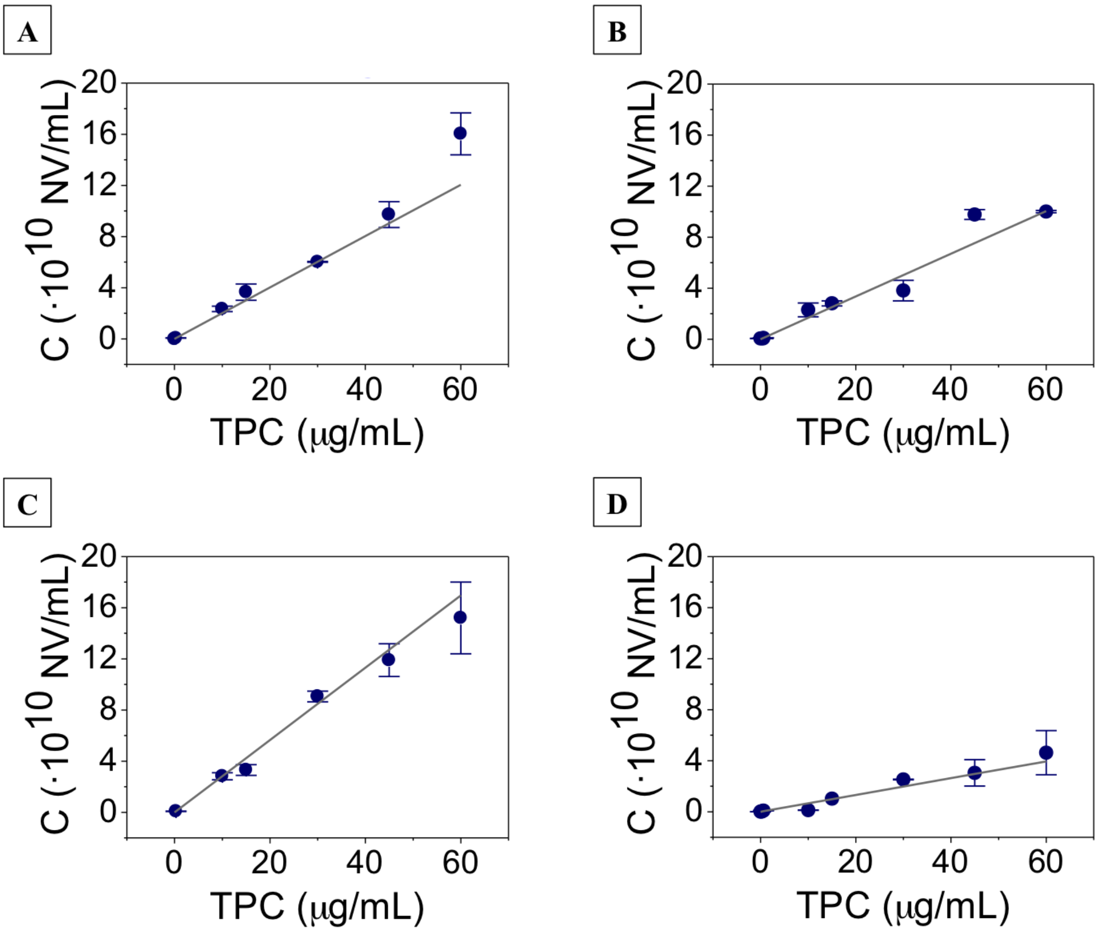

S 2: Calibration curves of nanovesicle concentration (C), obtained from the total area of NTA size distributions at different TPC concentrations. Linear dependence of the total protein concentration (TPC, expressed as μg/mL) on nanovesicle concentration (C, expressed as NV/mL)¡Error! Marcador no definido. (A) For SSTR2 carrying nanovesicles we found that the experimental data are correctly fitted by the equation $C(NVmL^{-1}) = (20.089 \pm 0.094) \cdot 10^{8} \cdot TPC(\mu gmL^{-1})$. (B) For c-myc-OR7D4-carrying nanovesicles we found that the experimental data are correctly fitted by the equation $C(NVmL^{-1}) = (16.743 \pm 0.103) \cdot 10^{8} \cdot TPC(\mu gmL^{-1})$. (C) For c-myc-OR1740 carrying nanovesicles we found that the experimental data are correctly fitted by the equation $C(NVmL^{-1}) = (27.877 \pm 0.967) \cdot 10^{8} \cdot TPC(\mu gmL^{-1})$. (D) For c-myc-OR1740 carrying nanovesicles (different expression batch) used to perform deposition onto functionalized gold surfaces we found that the experimental data are correctly fitted by the equation $C(NVmL^{-1}) = (6.581 \pm 0.066) \cdot 10^{8} \cdot TPC(\mu gmL^{-1})$

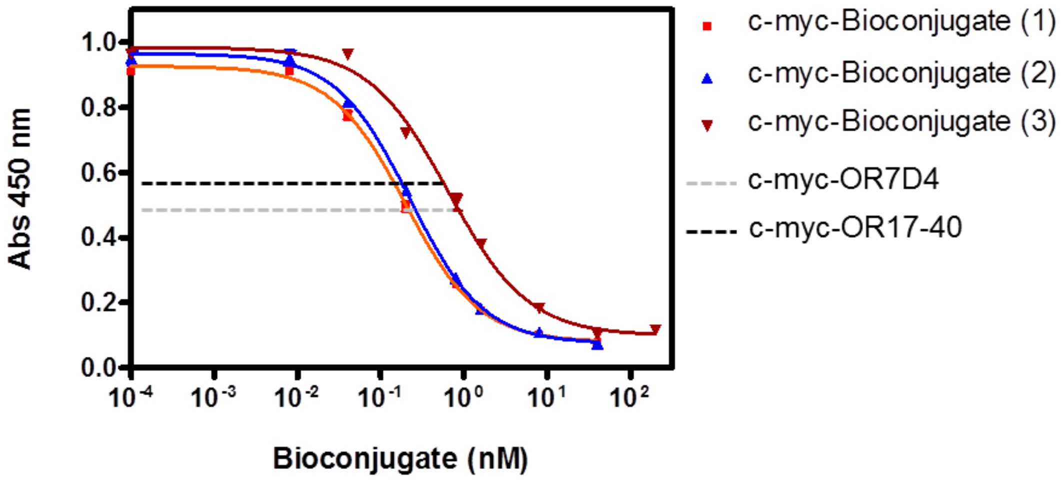

S 3: Inhibition curves of the three c-myc-Bioconjugates (analytes) at the concentration of SSTR2 carrying nanovesicles $7.03 \cdot 10^{10}$ NV/mL (35 µg/mL). The horizontal lines interpolated in the calibration curve mark the absorbance of the c-myc-OR17-40-carrying nanovesicles (black) and c-myc-OR7D4-carrying NVs (light grey) at the corresponding concentration ($9.8 \cdot 10^{10}$ NV/mL and $5.9 \cdot 10^{10}$ NV/mL, respectively). Calibration curves are built using three-well replicated and fitted with a four-parameter equation.

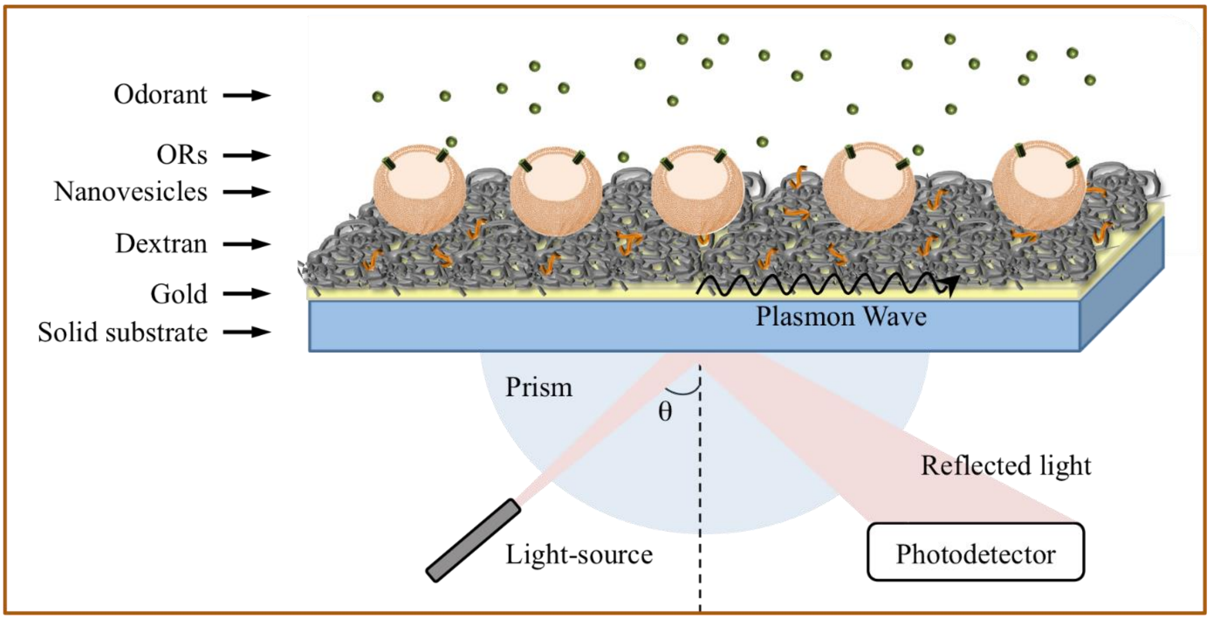

S 4: Schematic of the SPR setup showing the L1 sensor chip with the immobilized NVs.

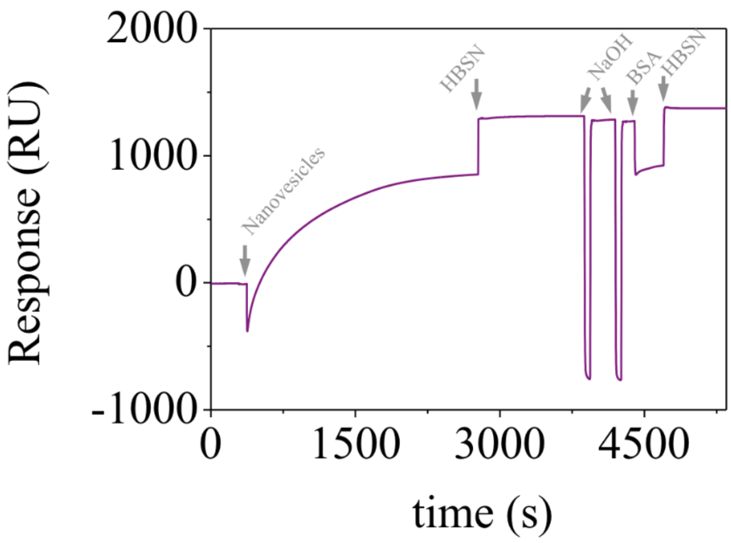

S 5: Typical Biacore sensorgram profile for NV carrying (in this case) the OR17-40 receptor (concentration = 15 µg/mL, t = 1800 s) and BSA (t = 300 s) immobilization onto a L1 chip. Analogous profiles were obtained for NVs carrying the SSTR2 receptor and NVs carrying the c-myc-OR7D4 receptor.

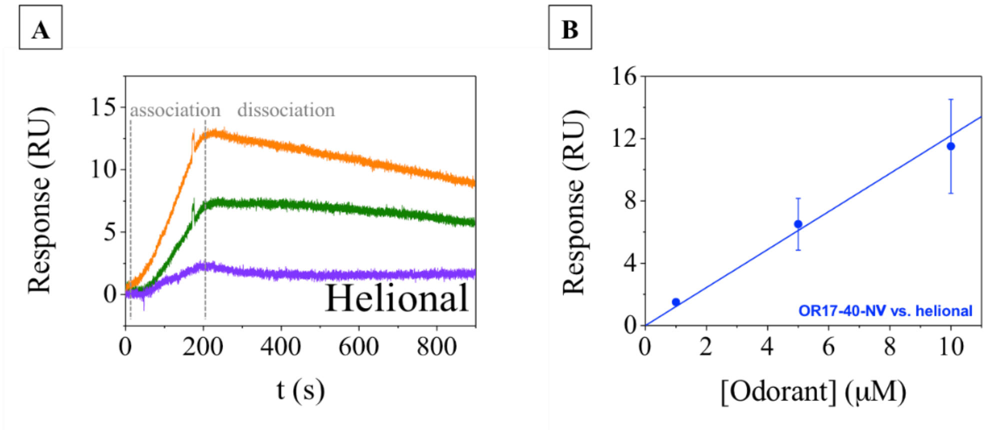

S 6: (A) Double-referenced SPR sensorgrams obtained from/in flowing solutions of helional at 1 µM (violet), 5 µM (green) and 10 µM (orange) onto a L1 chip with OR17-40-NV (flow rate: 60µL/min). (B) SPR response of OR17-40-NV vs. helional concentration (dots). The corresponding linear fit gives: y =1.21· x (solid line). In both plots the control nanovesicles used were OR7D4-NV.

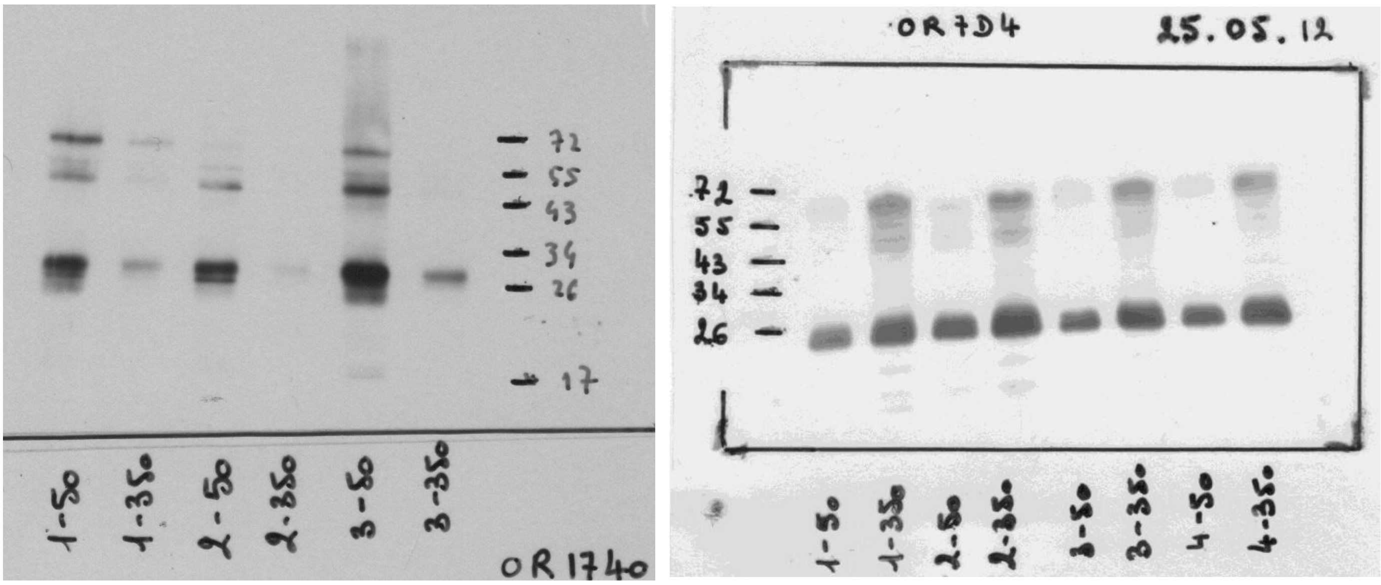

S 7 (A) Western Blot of eluates of purified c-myc-OR17-40, and (B) Western Blot of eluates of c-myc-OR7D4, after solubilization of membrane fractions of Saccharomyces cerevisiae. In each case, 450mg of c-myc-OR membrane fractions were solubilized using FC14 at 50 or 350 CMC.

Table S1. This table shows the features of the ELISA assay and its sensitivity at specific NV concentrations based on a four-parameter fitting. The assay features are repeated at each different NV concentration (data not shown). Each curve was built using three-well replicates.

Table 1: Features of the ELISA assay corresponding to Fig. 3.

	c-myc-Bioconj. (1)[a,b]	c-myc-Bioconj. (2)[a,b]	c-myc-Bioconj. (3)[a,b]
**A_{max}**	0.927 ± 0.008	0.965 ± 0.010	0.985 ± 0.019
**A_{min}**	0.078 ± 0.008	0.074 ± 0.010	0.098 ± 0.014
**IC_{50} (nM)**	0.198 ± 0.020	0.222 ± 0.027	0.646 ± 0.042
**Slope**	-0.975 ± 0.038	-0.979 ± 0.048	-0.903 ± 0.075
**R^2**	0.998 ± 0.016	0.997 ± 0.021	0.994 ± 0.029

[a] Coating antigen concentration used was 0.0125 mg/mL
[b] mAb dilution used was 1/160000

Table S2. Quantification of ORs/NV using the ELISA assay on nanovesicles using different protein bioconjugates as standard references.

	[TPC] (µg/mL)	[Vesicles] (NV/mL)	OR $NV^{-1, b}$			ORs $NV^{-1, c}$ $\bar{x}$	
			$^{3}C_1$-*BSA*	$^{4}C_1$-*CONA*	$^{1}C_1$-*HRP*	x_0	x_f
c-myc-OR1740	25	$7.0 \cdot 10^{10}$	1.04 ± 0.17	1.80 ± 0.34	--	1.42 ± 0.54	2.81±1.08
	30	$8.4 \cdot 10^{10}$	2.04 ± 0.10	4.69 ± 0.22	--	3.37 ± 1.88	
	35	$9.8 \cdot 10^{10}$	1.32 ± 0.01	2.86 ± 0.02	--	2.09 ± 1.08	
			3.79 ± 0.37	4.52 ± 0.37	5.53 ± 0.56	4.61 ± 0.87	
	45	$12.6 \cdot 10^{10}$	2.04 ± 0.02	3.81 ± 0.04	--	2.92 ± 1.25	
			1.46 ± 0.03	3.01 ± 0.05	2.93 ± 0.05	2.47 ± 0.87	
c-myc-OR7D4	25	$4.2 \cdot 10^{10}$	4.08 ± 0.41	7.48 ± 0.77	--	5.78 ± 2.41	5.56±2.35
	30	$5.0 \cdot 10^{10}$	4.35 ± 0.05	9.92 ± 0.12	--	7.14 ± 3.93	
	35	$5.9 \cdot 10^{10}$	2.74 ± 0.33	5.57 ± 0.60	4.04 ± 0.64	4.11 ± 1.41	
	45	$7.5 \cdot 10^{10}$	3.64 ± 0.19	6.77 ± 0.32	--	5.21 ± 2.21	

[a] *According to the linear correlation between the Total Protein Concentration (TPC, µg mL^{-1}) and the nanovesicle concentration (NV mL^{-1}) a ratio of 3.6 x 10^{-4} pg protein/ NV for OR1740-NVs and a ratio of 5.9 x 10^{-4} pg protein/NV for OR7D4-NVs were estimated (see S2). Results were obtained on different days.*

[b] *Concentration of ORs per nanovesicle resulting from interpolating the immunochemical response of each solution on the corresponding standard curve of each bioconjugate. Calculations were performed as described in a previous publication.*

[c] *Concentration of ORs per nanovesicle calculated as the measurements average using the different bioconjugates as standards for each TPC (x_0) and taking into consideration all measurements made at different TPC (xf),*

Table S2 shows the concentration of ORs/NV corresponding to each interpolated absorbance at each different bioconjugate calibration curve. The number of receptors per NV was obtained by dividing the number of receptors per mL by the corresponding number of NV per unit volume, as determined by Nanoparticle Tracking Analysis (NTA).
